# Clinically Relevant Plasmid-Host Interactions Indicate that Transcriptional and Not Genomic Modifications Ameliorate Fitness Costs of *Klebsiella pneumoniae* Carbapenemase-Carrying Plasmids

**DOI:** 10.1128/mBio.02303-17

**Published:** 2018-04-24

**Authors:** Michelle M. C. Buckner, Howard T. H. Saw, Rachael N. Osagie, Alan McNally, Vito Ricci, Matthew E. Wand, Neil Woodford, Alasdair Ivens, Mark A. Webber, Laura J. V. Piddock

**Affiliations:** a Antimicrobials Research Group, Institute of Microbiology & Infection, College of Medical & Dental Sciences, University of Birmingham, Birmingham, United Kingdom; b Institute of Microbiology and Infection, College of Medical & Dental Sciences, University of Birmingham, Birmingham, United Kingdom; c National Infection Service, Public Health England, Porton Down, United Kingdom; d Antimicrobial Resistance and Healthcare Associated Infections (AMRHAI) Reference Unit, Public Health England, London, United Kingdom; e Centre for Immunity, Infection and Evolution, University of Edinburgh, Edinburgh, United Kingdom; Louis Stokes Veterans Affairs Medical Center

**Keywords:** fitness, *Klebsiella pneumoniae* carbapenemase (KPC), genome, pKpQIL, plasmid, transcriptome

## Abstract

The rapid dissemination of antimicrobial resistance (AMR) around the globe is largely due to mobile genetic elements, such as plasmids. They confer resistance to critically important drugs, including extended-spectrum beta-lactams, carbapenems, and colistin. Large, complex resistance plasmids have evolved alongside their host bacteria. However, much of the research on plasmid-host evolution has focused on small, simple laboratory plasmids in laboratory-adapted bacterial hosts. These and other studies have documented mutations in both host and plasmid genes which occur after plasmid introduction to ameliorate fitness costs of plasmid carriage. We describe here the impact of two naturally occurring variants of a large AMR plasmid (pKpQIL) on a globally successful pathogen. In our study, after pKpQIL plasmid introduction, no changes in coding domain sequences were observed in their natural host, Klebsiella pneumoniae. However, significant changes in chromosomal and plasmid gene expression may have allowed the bacterium to adapt to the acquisition of the AMR plasmid. We hypothesize that this was sufficient to ameliorate the associated fitness costs of plasmid carriage, as pKpQIL plasmids were maintained without selection pressure. The dogma that removal of selection pressure (e.g., antimicrobial exposure) results in plasmid loss due to bacterial fitness costs is not true for all plasmid/host combinations. We also show that pKpQIL impacted the ability of K. pneumoniae to form a biofilm, an important aspect of virulence. This study used highly relevant models to study the interaction between AMR plasmids and pathogens and revealed striking differences from results of studies done on laboratory-adapted plasmids and strains.

## IMPORTANCE

Antimicrobial resistance is a serious problem facing society. Many of the genes that confer resistance can be shared between bacteria through mobile genetic elements, such as plasmids. Our work shows that when two clinically relevant AMR plasmids enter their natural host bacteria, there are changes in gene expression, rather than changes to gene coding sequences. These changes in gene expression ameliorate the potential fitness costs of carriage of these AMR plasmids. In line with this, the plasmids were stable within their natural host and were not lost in the absence of selective pressure. We also show that better understanding of the impact of resistance plasmids on fundamental pathogen biology, including biofilm formation, is crucial for fighting drug-resistant infections.

## INTRODUCTION

Plasmids consist of extrachromosomal DNA, and, although they are not essential, once acquired, they can become necessary for bacterial survival. Plasmids can confer beneficial traits, such as antimicrobial resistance (AMR). The worldwide dissemination of many carbapenemase genes and other clinically relevant beta-lactamase genes is largely due to conjugative plasmids. One such carbapenemase, the Klebsiella pneumoniae carbapenemase (KPC), was first detected in the United States and then around the world (1, 2). So far, in the United States, most K. pneumoniae outbreak isolates have been of sequence type 258 (ST258) and most have carried *bla*_KPC_ (3–5). The outbreak strains in the United States were very similar to an outbreak strain in Israel which carried *bla*_KPC-3_ (3, 6, 7). The success of K. pneumoniae ST258 is intricately linked with the success of *bla*_KPC_ (4, 8, 9). The plasmid most often associated with carbapenem resistance in ST258 isolates in Israel was named pKpQIL and was a conjugative 113,637-bp IncFII-like plasmid with *bla*_TEM-1_ and *bla*_KPC-3_ β-lactamase genes (10, 11). The *bla*_KPC_ gene is organized within an insertion of the Tn*4401* transposon (12, 13). The Tn*4401* transposon has likely contributed to the success of *bla*_KPC_ and can mediate transposition of *bla*_KPC_ into new genetic contexts, including new plasmids and chromosomes of the associated hosts (14, 15). Multiple isoforms of Tn*4401* have been identified. One study found five of the seven isoforms present in a collection of ST258 isolates (5). The most predominant *bla*_KPC_-carrying plasmids among ST258 isolates belonged to the IncFII_K2_-like group, which includes pKpQIL (16). Together, these data provide further evidence for the strong link between the success of IncF plasmids such as pKpQIL and the presence of Tn*4401*, *bla*_KPC_, and ST258. Sequencing of pKpQIL showed that it was stable during the 2-year Israeli outbreak (10). pKpQIL-like plasmids have been found in outbreaks, predominantly caused by K. pneumoniae, in countries such as Poland, Italy, Greece, United States, South Korea, and the Czech Republic (17–23). A recent study of 36 European countries detected KPC genes in 45% of carbapenemase-producing K. pneumoniae strains, making them the most frequently observed carbapenemase genes (24). Other carbapenemase genes identified in K. pneumoniae included the OXA-48-like, NDM, and VIM genes (24). Superspreading patients carrying carbapenemase-producing K. pneumoniae in health care environments, along with travel-associated acquisition, complicate control measures (25, 26). In some cases, infection control measures have been implemented and have been effective at controlling the spread of KPC-producing bacterial outbreak strains (see, e.g., references 27, 28, 29, and 30). Unfortunately, in other cases, outbreaks of infections by KPC-producing organisms have proven difficult to eradicate (15, 31).

Sequencing of *bla*_KPC_-positive plasmids in the United Kingdom (32) revealed some with high similarity to pKpQIL found in Israel (11). A dominant United Kingdom variant, termed pKpQIL-UK, was found in K. pneumoniae (33). A further variant, pKpQIL-D2, was isolated from K. pneumoniae, Escherichia coli, and *Enterobacter* species during a United Kingdom outbreak (33). Both pKpQIL-UK and pKpQIL-D2 have the *bla*_KPC-2_ gene located within the Tn*4401a* isoform (29, 33). Plasmid pKpQIL-D2 differs from most pKpQIL-like plasmids due to a substituted region; a 19.5-kb region of pKpQIL-UK has been replaced with a 17.6-kb region in pKpQIL-D2 (33). However, little is known about the impact of this substitution on the biology of the plasmid or the host bacteria. The variant regions between pKpQIL-UK and pKpQIL-D2 carry genes associated with drug resistance, mobile elements, a replicon, and plasmid partitioning (33).

Despite the global success of plasmids like pKpQIL and the difficulties associated with eradicating KPC-producing organisms, in general terms, plasmid persistence and survival within a bacterial population are considered to represent a paradox (34–36). Plasmids can impose a fitness defect with respect to the host bacterium but can also provide advantages, such as antimicrobial resistance genes (AGR). Plasmid success depends upon a balance of multiple factors, including conjugation rate (horizontal gene transfer), segregation error rate (vertical gene transfer), postsegregation killing, fitness cost(s), and compensatory mutations which can arise to reduce fitness cost(s) (34, 37, 38). A recent study demonstrated that even for plasmids that are costly with respect to fitness, conjugation can be sufficient in the absence of selective pressure to maintain a plasmid within a population (34). Not all plasmid-host combinations result in fitness costs, and plasmids maintained in certain well-adapted hosts may provide a plasmid reservoir (39). Plasmids encoding extended-spectrum β-lactamases can even increase the virulence and biofilm potential of E. coli strains, thus improving their adaptation to specific conditions (40). When fitness costs are associated with plasmid carriage, both mathematical models and experimental evidence support their amelioration by compensatory mutations (37, 39, 41–44). Compensatory mutations have been identified on plasmids, such as a mutation identified in a replication initiation protein gene, *trfA1* (IncP-1 plasmid), which reduced association with host helicase to ameliorate fitness costs (44). Compensatory mutations have also been identified in the bacterial chromosome, for example, in the *gacA gacS* two-component regulatory system, which modified expression of multiple genes (45). Intentional use of a costly plasmid in Pseudomonas aeruginosa revealed that compensatory mutations contributed to plasmid persistence (41, 46). These compensatory mutations were in putative helicase and kinase genes and were shown to reduce the transcriptional changes induced by plasmid carriage (41). Similarly, another study using *Pseudomonas* found that key compensatory mutations occurred in response to plasmid carriage in helicase and RNA polymerase genes (38). These mutations also increased the permissiveness of bacterial strains with respect to other plasmids (38). That study and other studies demonstrated that mobile genetic elements can impact the persistence and fitness of other independent mobile genetic elements (41, 42).

The Review on Antimicrobial Resistance acknowledged the increasing concerns of mortality and morbidity associated with AMR K. pneumoniae (47). The global priority list of AMR bacteria also includes carbapenem-resistant *Enterobacteriaceae*, including ST258/pKpQIL, as a critical priority (48). The global spread of pKpQIL-like plasmids and their host strains suggests that, once acquired, the plasmids are stable, confer little or no fitness cost to the host bacterium, and may even be beneficial. To determine the impact on the K. pneumoniae chromosome and plasmid, we investigated the impact of pKpQIL-UK and pKpQIL-D2 on pKpQIL-naïve K. pneumoniae strains using whole-genome sequencing (WGS) and RNA sequencing to determine the effect of plasmid carriage on global gene expression. The impact of either plasmid on growth, biofilm, and virulence was also determined. Plasmid fitness was assessed by measuring plasmid transfer, plasmid stability, and pairwise competition.

## RESULTS

### Whole-genome sequencing.

We hypothesized that compensatory mutations would occur in response to plasmid acquisition in the K. pneumoniae genome. Therefore, WGS was carried out for two K. pneumoniae strains, namely, an ST258 representative which is frequently associated internationally with pKpQIL (11, 22) and Ecl8, which is a widely used strain of this species (49, 50), each carrying either pKpQIL-UK or pKpQIL-D2. The ST258 representative isolate was a gift from B. Kreiswirth and was chosen because it is a carbapenem-susceptible isolate which did not contain a pKpQIL-like plasmid. Ecl8 belongs to the ST375 lineage of K. pneumoniae, which is phylogenetically distinct from ST258, with no shared MLST alleles. The sequences obtained from ST258 and Ecl8 carrying pKpQIL-UK or pKpQIL-D2 were compared with their respective pKpQIL-free isoforms. During strain construction, plasmids and hosts were grown together for 10 to 14 days prior to WGS (ca. 70 to 80 generations), during which time plasmid presence, strain identity, and PCR data were used to confirm transconjugants. After comparison of our pKpQIL-free ST258 strain, ST258/pKpQIL-UK (plasmid accession no. KY798507) and ST258/pKpQIL-D2 (plasmid accession no. KY798506), we were unable to detect any nonsynonymous single nucleotide polymorphisms (SNPs) in coding sequences of the chromosomes of either the ST258 or Ecl8 plasmid-carrying strains. The relatively small numbers of SNPs detected in the two strains were concentrated in intergenic regions, with parallelism observed in regions undergoing mutation in a given strain regardless of the plasmid present (see Table S1 in the supplemental material). For example, mutations were identified in the intergenic region downstream of *lrpC* in all four plasmid-containing isolates (Table S1). We observed a higher number of mutations in Ecl8 than in ST258.

10.1128/mBio.02303-17.4 TABLE S1  Single nucleotide polymorphisms found in chromosomes of K. pneumoniae strains carrying either pKpQIL-UK or pKpQIL-D2. Download TABLE S1, XLSX file, 0.01 MB. Copyright © 2018 Buckner et al. 2018 Buckner et al. This content is distributed under the terms of the Creative Commons Attribution 4.0 International license.

The plasmid WGS data revealed a few key differences. First, the pKpQIL-D2 plasmid in this study had two frameshift mutations compared with the published sequence (33). Second, in Ecl8, pKpQIL-D2 acquired five mutations, all in a mobile element, located within the 17.6-kb variable region (33). Third, the missense mutation in pKpQIL-UK was found only in ST258 and not Ecl8. Overall, pKpQIL-UK had fewer SNPs than pKpQIL-D2.

We wanted to determine if other (non-pKpQIL) plasmids were present in our strains. Ecl8 is known to have a large (206,152-kb) plasmid (50). PlasmidFinder identified an IncF1 replicon in the ST258 strain and an IncP replicon in Ecl8. Bandage was used to visualize de Bruijn graphs of the WGS data to determine if plasmid sequences were present in the pKpQIL-free ST258 isolate. Neither provided unambiguous evidence for the presence of plasmid contigs in ST258. Therefore, we were unable to determine from our data if other plasmids were present in the ST258 strain. However, our mapping analysis was performed on the entire genetic content of our parent ST258 strain. Therefore, even if other plasmids were present, and if changes in their sequences occurred as a result of the presence of pKpQIL, the mapping analysis would have identified any such substitution events. None of the substitutions detected in our analysis showed evidence of being plasmid associated. Therefore, our data support the hypothesis that no changes to the existing plasmid content occurred as a result of pKpQIL-D2/UK carriage.

**FIG 1:**
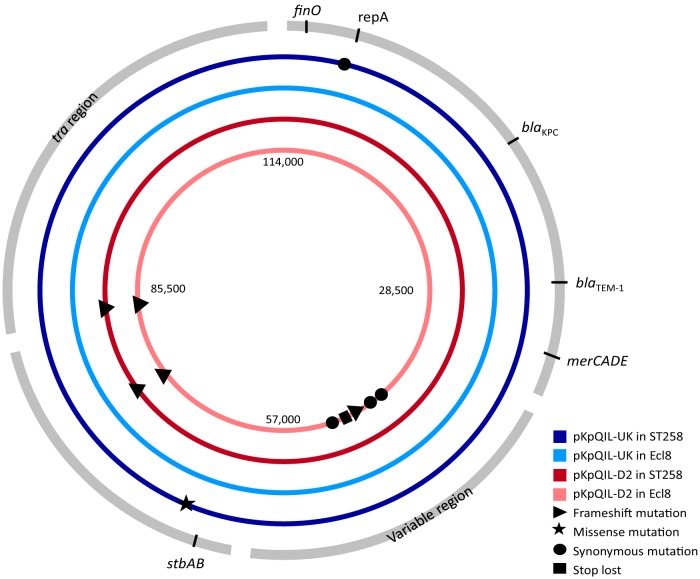
Diagrammatic representation of SNPs found in pKpQIL-UK in host strain ST258 (dark blue) and Ecl8 (light blue) and in pKpQIL-D2 in ST258 (dark red) and Ecl8 (pink). Approximate locations of regions of interest are labeled and indicated in the outer gray circle.

Comparison of pKpQIL sequences with reference sequences (33) revealed five SNPs (Fig. 1; see also Table S2). pKpQIL-UK in Ecl8 had no SNPs compared with ST258/pKpQIL-UK KY798507. pKpQIL-UK in ST258 had one missense mutation (L106S) in a single-stranded DNA binding protein. pKpQIL-D2 had two frameshift mutations in both ST258 and Ecl8 compared with ST258/pKpQIL-D2 KY798506, i.e., one deletion in a potential type I restriction enzyme and one in a hypothetical protein. In addition, pKpQIL-D2 in Ecl8 had five mutations within a 59-bp segment located within the variable region (bp 43737 to 43796).

10.1128/mBio.02303-17.5 TABLE S2  Single nucleotide polymorphisms identified in the pKpQIL-UK and pKpQIL-D2 plasmids used in this study (in either ST258 or Ecl8), compared to the KY798507 and KY798506 reference sequences, respectively. Download TABLE S2, XLSX file, 0.01 MB. Copyright © 2018 Buckner et al. 2018 Buckner et al. This content is distributed under the terms of the Creative Commons Attribution 4.0 International license.

### pKpQIL-UK and pKpQIL-D2 alter gene expression of K. pneumoniae ST258.

**FIG 2:**
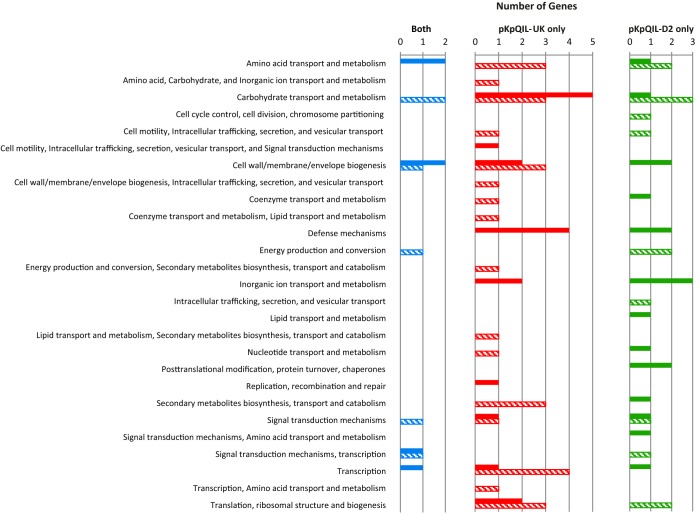
Gene expression changes in K. pneumoniae ST258 following acquisition of pKpQIL-UK or pKpQIL-D2, categorized by COG. Blue, genes altered in both ST258/pKpQIL-UK and ST258/pKpQIL-D2; red, genes altered in ST258/pKpQIL-UK but not in ST258/pKpQIL-D2, green, genes changed in ST258/pKpQIL-D2 but not in ST258/pKpQIL-UK; solid bars, upregulated genes; hashed bars, downregulated genes. Genes with unknown functions were not included.

RNA sequencing revealed that expression of 16 chromosomal genes was altered by carriage of both pKpQIL-D2 and pKpQIL-UK and that none were associated with alterations observed in intergenic regions of the chromosome (Table S3). In all 16 cases, expression was concordantly altered in ST258/pKpQIL-D2 and ST258/pKpQIL-UK compared to pKpQIL-free ST258. These 16 genes represent the impact of the core pKpQIL-like plasmids on ST258. Genes were categorized based on classifications of clusters of orthologous groups (COG) and were involved in processes such as amino acid and carbohydrate transport and metabolism, cell wall/membrane/envelope biogenesis, energy production and conversion, signal transduction, and transcription (Fig. 2, blue bars).

10.1128/mBio.02303-17.6 TABLE S3  Chromosomal genes with altered expression in both pKpQIL-UK-carrying ST258 and pKpQIL-D2-carrying ST258, compared to pKpQIL-free ST258. Genes were categorized based on Genoscope COG classifications. Download TABLE S3, XLSX file, 0.01 MB. Copyright © 2018 Buckner et al. 2018 Buckner et al. This content is distributed under the terms of the Creative Commons Attribution 4.0 International license.

In addition to core gene expression changes, compared with pKpQIL-free ST258, 63 genes were differentially expressed in ST258/pKpQIL-UK but were not altered in ST258/pKpQIL-D2 (Table S4). The genes belonged to various COG classifications (Fig. 2, green bars). The largest change was a 10-log-fold increase in expression of chromosomally carried genes *hsdM* and *hsdR* (*hsdMR*) (adjusted *P* = 3.4 × 10^−8^ and 8.7 × 10^−7^, respectively), encoding a restriction modification (RM) system, along with a 5-log-fold increase (adjusted *P* = 2.4 × 10^−9^) in expression of a putative transposase gene (KPN_00960) at a location adjacent to *hsdR*. Levels of expression of genes *acrA* and *acrB* (*acrAB*), encoding components of a multidrug resistance efflux pump, were increased (0.84-log-fold increase [adjusted *P* = 7.2 × 10^−6^] and 0.93-log-fold increase [adjusted *P* = 1.2 × 10^−6^], respectively), while *marR* expression was decreased (0.84-log-fold change; adjusted *P* = 0.011). The latter gene encodes a TetR repressor that impacts *acrAB* expression (51). Expression of *ydgF*, encoding a multidrug transporter belonging to the small multidrug resistance (SMR) superfamily, was increased by 0.75-log-fold (adjusted *P* = 0.031). However, no difference was detected in the MICs of SDS (>4,096 mg/liter), deoxycholate (>512 mg/liter), and bile salts (>4,096 mg/liter), which are reported substrates of YdgF (52). Expression levels of genes encoding proteins involved in copper sensing (*cusS*) and transport (*cusC*) were reduced (Table S4). However, the MICs of copper(II) sulfate (CuSO_4_) for ST258 alone or carrying either plasmid were the same (4 mM), and growth kinetics in CuSO_4_ did not reveal any advantage for the pKpQIL-UK-carrying strain. There was also a reduction in expression of *potG*, encoding the ATPase for the polyamine putrescine transport system (53).

10.1128/mBio.02303-17.7 TABLE S4  Genes with altered expression in ST258/pKpQIL-UK compared to pKpQIL-free ST258 or in ST258/pKpQIL-D2 compared to pKpQIL-free ST258. Gene categorization was performed on the basis of Genoscope COG classifications. Download TABLE S4, XLSX file, 0.02 MB. Copyright © 2018 Buckner et al. 2018 Buckner et al. This content is distributed under the terms of the Creative Commons Attribution 4.0 International license.

Carriage of pKpQIL-D2 resulted in differential expression of 38 genes (Table S4), all of which were unaffected by carriage of pKpQIL-UK. The genes with significantly altered expression in ST258/pKpQIL-D2 compared with pKpQIL-free ST258 belonged to several COG classifications (Fig. 2, red bars). These included a 1.8-log-fold reduction (adjusted *P* = 0.015) in *eutJ*, encoding a putative chaperone in ethanolamine utilization (54). Reductions of 1.7-log-fold and 1.9-log-fold (adjusted *P* = 0.024 and 0.022, respectively) were seen in the levels of expression of *hycFD*, involved in anaerobic fermentative growth (55). Expression of *mreB*, encoding an actin homologue important for cell elongation and peptidoglycan synthesis (56), was reduced by 0.2-log-fold (adjusted *P* = 0.044). There was a 0.25-log-fold increase (adjusted *P* = 0.017) in expression of penicillin-binding protein 3 gene *ftsI*, which is negatively regulated by MreB and is required for septation (57, 58). Expression of *tolC* was increased by 0.4-log-fold (adjusted *P* = 0.011). TolC is an outer membrane channel required for efflux through multiple efflux pumps, including AcrAB and OqxAB (59–62). The presence of pKpQIL-D2 resulted in an increase in expression of *oqxB* of 0.5-log-fold (adjusted *P* = 0.025), along with a 0.6-log-fold increase (adjusted *P* = 0.045) in expression of the gene encoding its regulator, RarA.

As RNA sequencing revealed an increase in expression of *oqxB* in ST258/pKpQIL-D2 and as OqxAB is known to efflux olaquindox, the susceptibility of the strains to olaquindox was determined. The MICs for the strains were 256 mg/liter (ST258), 128 mg/liter (ST258/pKpQIL-UK), and 256 mg/liter (ST258/pKpQIL-D2), indicating no significant differences between strains.

### Expression levels of plasmid genes differed between pKpQIL-UK and pKpQIL-D2.

Next, we examined gene expression from pKpQIL-UK and pKpQIL-D2 in ST258. Fifty-four identical genes were expressed from both plasmids (Table S5), including genes involved in replication and transmission, transposon-associated genes, and genes associated with toxin-antitoxin systems. *bla*_KPC_ and *bla*_TEM_ were highly expressed from both plasmids. The data revealed moderate expression of *merAC* heavy metal resistance genes and of three genes encoding modifications of host defense mechanisms. Eleven genes with high DNA sequence similarity, located predominantly within the variable region, in the two plasmids were also expressed (Table S5), including genes encoding DNA modification, recombinase, replication, segregation, stability, transmission, and modifications to host defense.

10.1128/mBio.02303-17.8 TABLE S5  Core genes which are expressed from both pKpQIL-UK and pKpQIL-D2 in ST258. Sequences of each gene on both plasmids are either 100% identical (top portion) or highly (70% to 99.9%) similar. Genes are categorized based on function. Boldface data indicate a greater than 1-fold difference in expression between pKpQIL-UK and pKpQIL-D2. Download TABLE S5, XLSX file, 0.02 MB. Copyright © 2018 Buckner et al. 2018 Buckner et al. This content is distributed under the terms of the Creative Commons Attribution 4.0 International license.

**FIG 3:**
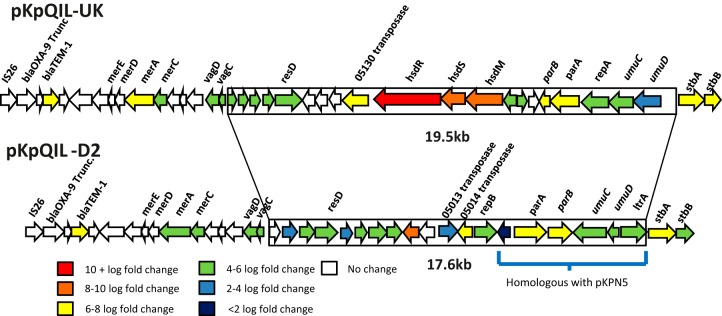
Comparison of the substituted fragments of pKpQIL-UK and pKpQIL-D2. A 19.5-kb region on pKpQIL-UK was replaced with a 17.6-kb fragment to form pKpQIL-D2. The fragment on pKpQIL-D2 (blue bracket) shares 98% DNA sequence identity with plasmid pKPN5 from K. pneumoniae MGH78578. Genes are color coded based on expression (log fold changes) in K. pneumoniae ST258, determined by RNA sequencing.

Thirteen genes unique to pKpQIL-UK and 14 genes unique to pKpQIL-D2 were expressed in ST258 (Table S6). The unique genes expressed by pKpQIL-UK included one gene encoding a factor involved in replication, one transposase gene, two genes involved in plasmid partitioning, three genes involved in RM systems, and six genes of unknown function. For pKpQIL-D2, one gene encoding a factor involved in DNA modification, one gene involved in replication, two transposon-related genes, two plasmid partitioning genes, and eight genes of unknown function were expressed. These uniquely expressed genes were found within the variable regions (Fig. 3).

10.1128/mBio.02303-17.9 TABLE S6  Genes which are carried by and are unique to pKpQIL-UK or pKpQIL-D2. Download TABLE S6, XLSX file, 0.01 MB. Copyright © 2018 Buckner et al. 2018 Buckner et al. This content is distributed under the terms of the Creative Commons Attribution 4.0 International license.

Our data show that genes involved in modifying bacterial defense mechanisms were expressed by both plasmids. These included *ssb*, encoding an antirestriction protein which inhibits RM systems (63), and *psiA* and *psiB*, which are involved in inhibiting the SOS response and can be triggered by single-stranded DNA, e.g., during conjugation (64).

Three genes, *umuC*, *umuD*, and *resD*, were expressed from both plasmids and are located within the variable regions with highly similar sequences, although in slightly different positions (Table S5). We did not detect expression of four open reading frames (ORFs) from the variable region of pKpQIL-UK or of two ORFs from the variable region of pKpQIL-D2 (white arrows in Fig. 3), encoding hypothetical proteins. These data show that, under these conditions, most of the genes within the variable regions were expressed in K. pneumoniae ST258.

### In K. pneumoniae, Ecl8/pKpQIL-D2 outcompetes Ecl8/pKpQIL-UK.

**FIG 4:**
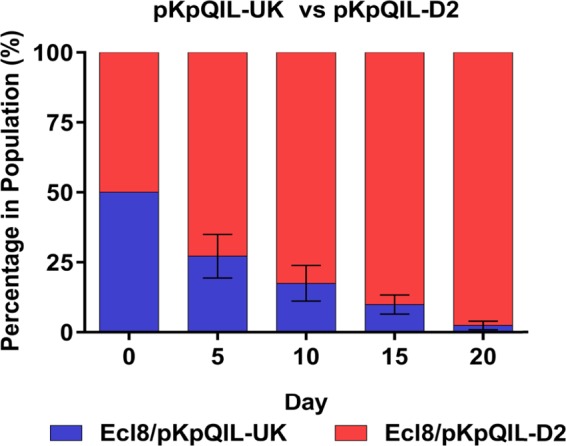
Pairwise competition of pKpQIL-UK (blue) versus pKpQIL-D2 (red) using rifampin-resistant K. pneumoniae Ecl8 as the host. Percentages of plasmid-carrying bacteria were recorded as means ± standard deviations of results from three independent experiments. The competition index value at the end of the experiment was 0.96.

To determine the impact of the variable regions on fitness, a pairwise competition assay was carried out to study plasmid carriage over 20 days. pKpQIL-like plasmids have been typically associated with K. pneumoniae ST258 (11, 22), and this strain would therefore have been ideal for competition experiments. However, the available ST258 strain was resistant to kanamycin and chloramphenicol, marker genes used for plasmid gene inactivation. We attempted to insert tellurite resistance gene *tpm* into the plasmid. However, despite repeated attempts, we were unable to successfully isolate plasmids with this insertion. Therefore, we used widely used K. pneumoniae strain Ecl8 (49, 50, 65, 66), which is susceptible to kanamycin. To differentiate pKpQIL-UK and pKpQIL-D2 from each other, *bla*_KPC_ in both plasmids was replaced with *aph*, conferring kanamycin resistance. To determine whether *aph* impacted persistence, two experiments were carried out with pKpQIL-UK versus pKpQIL-D2 *bla*_KPC_::*aph* and pKpQIL-UK *bla*_KPC_::*aph* versus pKpQIL-D2. Irrespective of which plasmid contained *aph*, pKpQIL-D2 outcompeted pKpQIL-UK (Fig. 4).

### Plasmid pKpQIL-UK transfers at a higher rate than pKpQIL-D2, but the two persist equally well.

There was no difference between the generation times of plasmid-free Ecl8 and ST258 or of the associated strains carrying the different plasmids (Table 1). Strikingly, pKpQIL-UK transferred at a higher frequency (ca. 18-fold, *P* < 0.05) than pKpQIL-D2 from their respective original K. pneumoniae clinical isolates (ST468/pKpQIL-UK, ST321/pKpQIL-D2) into ST258 (Table 1), but the level of transfer of pKpQIL-UK into Ecl8 was only 1.7-fold higher than that of pKpQIL-D2 (Table 1). When the original K. pneumoniae isolates with their respective plasmids, ST258 containing either plasmid, or Ecl8 containing either plasmid were grown over 20 days (ca. 140 generations) without antibiotic selection, the two plasmids persisted equally well in each of the host bacterial strains (see Fig. S1 in the supplemental material).

10.1128/mBio.02303-17.1 FIG S1  Percentages of pKpQIL-UK-carrying versus pKpQIL-D2-carrying cells over a period of 20 days without antibiotic selection. The persistence of plasmids pKpQIL-UK (blue) and pKpQIL-D2 (red) was assayed in K. pneumoniae (A) ST468/pKpQIL-UK and ST321/pKpQIL-D2, (B) ST258, and (C) Ecl8, all in LB broth without antibiotic selection. The percentages of cells which retained the plasmids were recorded as means ± standard deviations of results of three independent experiments. Download FIG S1, PDF file, 0.3 MB. Copyright © 2018 Buckner et al. 2018 Buckner et al. This content is distributed under the terms of the Creative Commons Attribution 4.0 International license.

**TABLE 1:** Summary of results of the fitness experiments for pKpQIL-UK-carrying and pKpQIL-D2-carrying K. pneumoniae

Host strain	Plasmid	Conjugation frequency^a^	Growth^b^	Persistence^c^	Biofilm result for indicated condition^d^	
					Plastic	Flow
ST258	pKpQIL-UK	3.3 × 10^−4^	32.9 ± 4.8	=	=	↑
	pKpQIL-D2	1.8 × 10^−5^	33.9 ± 5.0	=	=	↓
						
Ecl8^Rif^	pKpQIL-UK	1.57 × 10^−5^	31.8 ± 5.7	=	↓^e^	↓^e^
	pKpQIL-D2	9.13 × 10^−6^	33.9 ± 5.6	=	=	=

a Data represent results of conjugation from the original isolates (ST468/pKpQIL-UK and ST321/pKpQIL-D2) into ST258.

b Data represent generation times (minutes).

c Data represent results of comparisons of the host strain containing pKpQIL-UK to same strain containing pKpQIL-D2 (not in competition). =, no difference.

d Data represent results of comparisons to a strain with no plasmid. =, no difference; ↓, significantly lower; ↑, significantly higher.

e Biofilm experiments were performed at 22°C; no difference was seen at 37°C.

### The presence of plasmid pKpQIL-UK or plasmid pKpQIL-D2 altered host biofilm formation.

Biofilms can be important for bacterial survival and virulence, since the levels of K. pneumoniae biofilm production can be highly variable between strains (67, 68); however, the evidence concerning the impact of plasmids on biofilm production is conflicting (69–72). Therefore, we determined if the substituted region in pKpQIL-D2 conferred an advantage in biofilm formation compared to pKpQIL-UK (Table 1). On a plastic surface, both ST258/pKpQIL-UK and ST258/pKpQIL-D2 formed a biofilm as well as the plasmid-free ST258 did (Fig. S2a). However, Ecl8/pKpQIL-UK formed significantly less biofilm on plastic at 22°C than pKpQIL-free Ecl8 or Ecl8/pKpQIL-D2 (Fig. S2b). In a constant-flow/microfluidic-channel biofilm model, ST258/pKpQIL-UK had increased biofilm formation compared with pKpQIL-free ST258 (Fig. S2c). Conversely, ST258/pKpQIL-D2 formed less biofilm than ST258 (Fig. S2c). Under flow conditions, in Ecl8, pKpQIL-UK formed less biofilm than Ecl8 or Ecl8/pKpQIL-D2 (Fig. S2d).

10.1128/mBio.02303-17.2 FIG S2  Biofilm formation (a) on plastic for ST258 at 30°C and (b) on plastic for Ecl8 at 22°C. The impact of plasmids pKpQIL-UK (blue) and pKpQIL-D2 (red) on the ability of the hosts to form biofilm was investigated using the microtiter tray and crystal violet dye method. Percent fold change was recorded as means ± standard deviations of results of a minimum of three independent experiments. (c) Percentages of coverage by biofilm in microfluidic channel after 48 h under conditions of a constant flow of LB broth by pKpQIL-UK (blue)-carrying and pKpQIL-D2 (red)-carrying K. pneumoniae ST258 on the surface of the microfluidic channel were estimated using ImageJ software. The percentages of coverage relative to the parental strain were recorded as means ± standard deviations of results of three independent experiments. (d) Biofilm growth of Ecl8, Ecl8/pKpQIL-UK, and Ecl8/pKpQIL-D2 in a fluidic channel under conditions of constant flow at 37, 30, and 22°C. Images are representative of results of three independent experiments. Student’s *t* test was used to analyze significant changes which are shown with asterisks (*, *P* < 0.05). Download FIG S2, PDF file, 0.4 MB. Copyright © 2018 Buckner et al. 2018 Buckner et al. This content is distributed under the terms of the Creative Commons Attribution 4.0 International license.

### Neither plasmid altered K. pneumoniae virulence in Galleria mellonella.

The virulence of K. pneumoniae ST258 was determined in the G. mellonella model to establish if the substituted region in pKpQIL-D2 conferred a difference in virulence compared with ST258/pKpQIL-UK. No difference in survival was observed for G. mellonella infected by ST258/pKpQIL-UK, ST258/pKpQIL-D2, or pKpQIL-free ST258 (Fig. S3). However, while G. mellonella is a widely used model for virulence, it may not fully reflect the effects that occur in the mammalian host. Future work could use murine models to address the potential virulence impacts of the plasmids within the mammalian hosts.

10.1128/mBio.02303-17.3 FIG S3  Virulence assay of Galleria mellonella infected by K. pneumoniae ST258 carrying pKpQIL plasmid variants. Groups of 30 larvae were challenged with 1 × 10^5^ CFU of wild-type host ST258 (black) or ST258 carrying either pkpQIL-UK (blue) or pKpQIL-D2 (red). Percentages of G. mellonella that survived after infection were recorded at 24-h intervals for 5 days. The log rank (Mantel-Cox) test was used to analyze the differences in the survival rates of the two populations infected with plasmid-carrying K. pneumoniae ST258 compared with the wild-type host. Download FIG S3, PDF file, 0.3 MB. Copyright © 2018 Buckner et al. 2018 Buckner et al. This content is distributed under the terms of the Creative Commons Attribution 4.0 International license.

## DISCUSSION

In our study, the impact of a globally disseminated KPC plasmid (pKpQIL-UK) and a variant (pKpQIL-D2) upon K. pneumoniae was investigated. Bacterial fitness is a complex topic and can be broadly defined as the ability of bacteria to alter metabolism to adapt to environmental conditions, in order for their genetic traits to compete, survive, and reproduce within a host strain or to transfer to different hosts (73). Plasmid success is dependent on a variety of factors, including impact upon fitness, conjugation rate, and segregation error rate (34, 37). It is widely thought that plasmid acquisition incurs a fitness cost, resulting in plasmid-bearing bacteria being outcompeted by plasmid-free strains (74–76). Plasmid carriage has also been previously reported to confer lower growth rates (74, 77). Plasmids that are costly with respect to fitness can induce compensatory mutations on either the plasmid or chromosome to reduce the potential fitness defect of plasmid carriage (38, 41, 43, 46). In our study, the growth rates of K. pneumoniae strains ST258 and Ecl8 carrying either pKpQIL-UK or pKpQIL-D2 were indistinguishable from those of the pKpQIL-free host strains. We had previously made the same observation for *bla*_CTX-M-14_-carrying strains of *Enterobacteriaceae* (78). We found only a few intergenic mutations occurring in the genome of strains upon acquisition of pKpQIL. This is in accordance with a study of E. coli ST131 (79). However, none of the intergenic mutations observed in our data were directly associated with changes in gene expression, despite significant transcriptomic changes. The SNPs in the intergenic regions downstream of *lrpC* are interesting, as Lrp and Lrp homologues are global regulators of gene expression (80, 81). Previous reports have indicated that Lrp can positively regulate expression of *traJ* (in *Salmonella* plasmids pSLT and R100 and in E. coli plasmid pRK100), which mediates expression of the *tra* operon involved in plasmid conjugation (82–84).

Depending on the biofilm model used, relative to the pKpQIL-free hosts, the two plasmids variably affected the ability of K. pneumoniae ST258 and Ecl8 to form a biofilm. Biofilm formation is an important phenomenon, as most bacteria in the natural environment exist in biofilms (85), which play an important role in infection and AMR (76). Other studies have also found the biofilm formation characteristics of different K. pneumoniae strains to differ greatly (67, 68). Conflicting data associating plasmid presence and/or ability to conjugate with biofilm formation have been previously reported (40, 69–72) and may reflect the models used. The differences in biofilm formation that we observed between strains carrying pKpQIL-UK or pKpQIL-D2, such as the improved biofilm formation of ST258/pKpQIL-UK under flow conditions, may be important for biofilm formation on surfaces such as hospital sink drains, where biofilms contribute to bacterial and antibiotic resistance gene (ARG) dispersal (86, 87). Biofilms in these settings could provide a reservoir for both bacterial plasmids and ARGs. Surprisingly, none of the SNPs or transcriptomic changes identified in the pKpQIL-carrying strains were among those previously identified as important in biofilm formation.

The stability of large, low-copy-number plasmids within a bacterial population is facilitated by postsegregation killing and active plasmid-encoded partitioning mechanisms (88). Both plasmids carry plasmid maintenance genes, including genes *parA* and *parB* and genes *stbA* and *stbB* (11). When grown separately, both plasmids persisted within the population. However, in pairwise competition assays using isogenic hosts, pKpQIL-D2 conferred a reproducible advantage over pKpQIL-UK. The comparable growth rates of all strains negate the impact of growth, and since the two plasmids are of the same incompatibility group, the transfer of pKpQIL-D2 into pKpQIL-UK-carrying Ecl8 would be unlikely. Likewise, plasmid transfer following plasmid loss was unlikely, as the two plasmids persisted equally well. In addition, because pKpQIL-UK had a higher conjugation frequency than pKpQIL-D2, it was unlikely that conjugation caused pKpQIL-D2 to outcompete pKpQIL-UK. It is possible that differences between the donor clinical isolates contributed to the differences in conjugation frequency. Hardiman et al. have recently underscored the impact of strain differences on conjugation frequency (89). It is also plausible that pKpQIL-UK transmits to new hosts rapidly, allowing it to move within a population. Meanwhile, pKpQIL-D2 does not transmit as readily but, once inside a host, provides a fitness advantage in comparison to K. pneumoniae with pKpQIL-UK.

RNA sequencing revealed that expression of a small number (*n* = 16) of chromosomal genes was altered by the presence of either plasmid. These data suggest that both pKpQIL-UK and pKpQIL-D2 cause general perturbations in host bacterial pathways involved in processes such as metabolism, transport, cell wall/membrane maintenance, signaling, and transcription. These changes in expression may compensate for any impact that the plasmid has on the cell, thus reducing the fitness cost of each plasmid. In line with our observation of a core transcriptional response to pKpQIL, Shintani et al. found that, among three different strains of *Pseudomonas*, carriage of pCAR1 (an IncP-7 plasmid) resulted in the presence of a small set of core chromosomal genes with altered expression (90). Similarly to pKpQIL and pCAR1, a broad-host-range IncA/C plasmid (pAR060302) was found to consistently alter central-metabolism-related genes across different species (E. coli, Salmonella enterica serovar Newport, *S. enterica* servovar Heidelberg, S. enterica serovar Enteritidis, and Shewanella oneidensis), possibly to optimize energy flux for successful plasmid maintenance (91). Changes in gene expression in ST258/pKpQIL-UK included a 10-fold increase in the expression of type I RM genes *hsdM* and *hsdR*, the largest gene expression change in our data set. Chromosomal *hsdS* was not highlighted in our data. However, *hsdR*, *hsdS*, and *hsdM* were expressed from pKpQIL-UK. It is possible that the product of the plasmid *hsdS* gene is able to interact with the upregulated chromosomal *hsdM* and *hsdR* gene products, accounting for the lack of upregulation of the chromosomal *hsdS* gene. The reason for the upregulation of both chromosomal and plasmid-encoded type I RM systems in pKpQIL-UK but not in pKpQIL-D2 is unknown. However, RM systems can be a barrier to plasmid conjugation (92); therefore, this system may help prevent uptake of new plasmids or DNA, ensuring pKpQIL-UK survival.

The RNA sequencing data revealed the “core” plasmid genes expressed in ST258. These genes comprise those encoding products with functions that include replication, transmission, stability, recombination, toxin-antitoxin systems, antibiotic and heavy metal resistance, and DNA binding. Antibiotic resistance genes *bla*_TEM_ and *bla*_KPC_ were highly expressed by both plasmids, with the presence of the latter gene explaining the high level of resistance to beta-lactam and carbapenem antibiotics conferred by both plasmids. In total, approximately 60% of the predicted coding sequences of pKpQIL-UK and pKpQIL-D2 were expressed under the conditions tested here. In contrast, backbone genes of an IncA/C plasmid (pAR060302) were found to be relatively inactive (18% expressed), based on the gene expression levels seen under different culture conditions, with and without antibiotics (93). This difference could have been due to pKpQIL-like plasmids and *bla*_KPC_ being well adapted to ST258 K. pneumoniae host strains (4, 5, 8, 9). In addition, a substantial portion of pKpQIL is predicted to be involved in transmission (33), and our data demonstrate that multiple transmission genes were transcribed, which corresponds with the high conjugation frequency that we observed.

We were unable to detect any chromosomal SNPs within coding regions in our WGS data. However, sufficient changes at the transcriptional level may ameliorate fitness costs of the plasmid, and thus, no significant changes in the host genome occurred in our experiments. The strong associations among K. pneumoniae, pKpQIL, Tn*4401*, and *bla*_KPC_ may indicate that that plasmid and that host are well adapted to each other. This may explain why the combination of pKpQIL and K. pneumoniae is so globally successful (10, 17–23). One study used a pKpQIL-like *bla*_KPC-2_-carrying plasmid and moved it into a naive E. coli strain, where it caused only minimal fitness costs (94). It is also possible that long-term evolutionary experiments may show plasmid-host coevolution in coding regions of the genome. Many rearrangements have been identified in pKpQIL and pKpQIL-like plasmids in clinical isolates, but the association of *bla*_KPC_ with the Tn*4401* transposon is generally well maintained in *Klebsiella* (21, 22, 29, 95, 96). One study even reported rearrangements that resulted in the loss of the Tn*4401* transposon containing *bla*_KPC-3_ during the course of an infection (97). The purpose of our study was not to explore long-term evolutionary changes to plasmid structure; rather, it was to document the early events after plasmid acquisition which we found to occur through transcriptional modulation upon plasmid transfer. It is also possible that the ancestor of the ST258 strain used may have contained a pKpQIL or pKpQIL-like plasmid and then lost it prior to isolation. Similar events have been suggested previously for ST258 (4, 9). We have no evidence to support or refute this hypothesis. If the ancestor of the ST258 strain used carried and lost a pKpQIL-like plasmid, there may have been evolutionary adaptations to plasmid carriage which would not be identified in the present study. Another possibility is that a pKpQIL-like plasmid existed historically in an ancestor of ST258 and that the plasmid acquired the Tn4401 transposon carrying *bla*_KPC_ in the early 2000s. If this were the case, we would anticipate that the pKpQIL-like plasmids would be well adapted and would result in little or no fitness cost, as was seen in these experiments. However, we have no evidence to support or refute the idea of the historical presence of a *bla*_KPC_-free pKpQIL-like precursor in our strains. Furthermore, while this might have been possible, Bowers et al. found only 5 of most recent common ancestors among 167 isolates in clade 1, and 2 isolates might have harbored and then lost pKpQIL-like plasmids (4).

The widespread prevalence of *bla*_KPC_-carrying K. pneumoniae poses a serious threat to medical treatment. pKpQIL and its variants have played a significant role in the dissemination of KPC across bacterial sequence types and species (98). As *bla*_KPC_ confers resistance to and hence survival in the presence of carbapenem and most other beta-lactam antibiotics, the use of these antibiotics is a factor driving global dissemination. Since KPC-encoding plasmids, such as pKpQIL, cause such low fitness costs to the host and are stable within it, a reduction in the use of carbapenem antibiotics is unlikely to change plasmid prevalence. Our data indicate that these AMR plasmids persist in the absence of drug pressure, which emphasizes the importance of the diverse strategies needed to contain AMR, including rapid detection of resistant bacteria and effective infection prevention and control measures to limit transmission.

## MATERIALS AND METHODS

### Bacterial strains, plasmids, and growth conditions.

The plasmids, bacterial strains, and primers used in this study are listed in Table 2 and 3. The rifampin-resistant mutants of K. pneumoniae Ecl8 were selected as previously described (69). Plasmids with *bla*_KPC_::*aph* were generated as previously described (69, 99). Plasmids were transferred into new hosts by filter mating (69). PCR and DNA sequencing were used for verification. Throughout this study, “pKpQIL-free” refers to the state of the bacterial host before pKpQIL plasmid introduction. Bacteria were grown in MOPS (morpholinepropanesulfonic acid) minimal media for RNA sequencing experiments. Unless otherwise stated, all other experiments were carried out in LB media.

**TABLE 2:** Strains and plasmids used in this study^a^

Plasmid, clinical isolate, or strain	Description	Reference or source
Plasmids		
pKpQIL-UK	A *bla*_KPC-2_-carrying pKpQIL plasmid isolated in the United Kingdom	33
pKpQIL-D2	A pKpQIL-like plasmid isolated in the United Kingdom carrying *bla*_KPC-2_	33
pKD4	A plasmid carrying a kanamycin resistance cassette (*aph*)	J. A. Cole
		
Clinical isolates		
L27	K. pneumoniae ST321 carrying pKpQIL-D2 plasmid	N. Woodford
L33	K. pneumoniae ST468 carrying pKpQIL-UK plasmid	N. Woodford
		
Strains		
K. pneumoniae Ecl8	K. pneumoniae Ecl8	49
K. pneumoniae Ecl8^Rif^	Rifampin-resistant mutant of Ecl8; *rpoB* His537Leu	This study
K. pneumoniae Ecl8^Rif^/United Kingdom	Ecl8^Rif^ transconjugant carrying pKpQIL-UK	This study
K. pneumoniae Ecl8^Rif^/UK-aph	Ecl8^Rif^ transconjugant carrying pKpQIL-UK with inactivated *bla*_KPC_ gene	This study
K. pneumoniae Ecl8^Rif^/D2	Ecl8^Rif^ transconjugant carrying pKpQIL-D2	This study
K. pneumoniae Ecl8^Rif^/D2-aph	Ecl8^Rif^ transconjugant carrying pKpQIL-D2 with inactivated *bla*_KPC_ gene	This study
K. pneumoniae ST258	K. pneumoniae ST258 (Kp33636)	B. N. Kreiswirth
K. pneumoniae ST258/United Kingdom	ST258 transconjugant carrying pKpQIL-UK	This study
K. pneumoniae ST258/D2	ST258 transconjugant carrying pKpQIL-D2	This study
E. coli DH10B	F^−^ *mcrA* Δ(*mrr-hsdRMS*-*mcrBC*) φ80*lac*Z ΔM15 Δ*lacX74 recA1 endA1* *araD139* Δ(*ara*, *leu*)*7697 galU galK* λ^−^ *rpsL nupG tonA*	Invitrogen
E. coli SW105	E. coli carrying recombinase genes used in gene inactivation	107
E. coli 10418	E. coli NCTC10418	National Collection of Type Cultures (NCTC)

a UK, pKpQIL-UK plasmid; D2, pKpQIL-D2 plasmid; Rif, rifampin.

**TABLE 3:** Primers used in this study

Name	DNA sequence (5′ to 3′)^a^	Description	Source
KPCg-colpcrF	ATGTCACTGTATCGCCGTCT	Detection of the presence of *bla*_KPC_	This study
KPCg-colpcrR	TAGACGGCCAACACAATAGG		
			
KPCg-KO-F	CAACCTCGTCGCGGAACCATTCGCTAAACTCGAACAGGACTTTG(GTGTAGGCTGGAGCTGCTTC)	Inactivation of *bla*_KPC-2_ gene	This study
KPCg-KO-R	GCCAGTGCAGAGCCCAGTGTCAGTTTTTGTAAGCTTTCCG(GGGAATTAGCCATGGTCCAT)		
			
pQIL-F	CAGCATGACAGAATAGCGAGGCTT	Differentiation of pKpQIL-UK from -D2 plasmid	This study
pQIL-R	TACAAGGAGATGTGCCATGACCGT		
			
pMan-F	CTTACTGGCAAACTGTTGA	Differentiation of pKpQIL-D2 from -UK plasmid	This study
pMan-R	ATCCCGTGTGTTCAAAA		

a Sequences in parentheses are homologous to the *aph* gene cassette on plasmid pKD4.

### Growth kinetics.

The bacterial growth rate seen during the logarithmic phase was used to determine the fitness of the strain by the use of a FLUOstar Optima plate reader (BMG Labtech) as described previously (69). Bacterial generation times were calculated from the logarithmic phase of growth and were determined on three separate occasions as previously described (78). The value for the pKpQIL-free strain was compared with that for the plasmid-containing strain using Student’s *t* test, and the generation times were considered significantly different when the *P* value was <0.05.

### Determination of conjugation frequency.

The conjugation frequency of plasmids was determined by the use of their original clinical isolates as donors as previously described (69, 78). Ranges of donor/recipient ratios were tested using filter mating, and a ratio of 8:1 resulted in the highest conjugation frequency. This ratio was therefore used for the original ST468/pKpQIL-UK and ST321/pKpQIL-D2 isolates, which were incubated at 37°C for 3 h. Bacteria were removed, serially diluted, and plated on agar supplemented with selecting antibiotics and incubated overnight at 37°C. The plasmid conjugation frequency was calculated according to the following formula: the number of transconjugant colonies (in CFU per milliliter) divided by the ratio of conjugation (e.g., 8/1) multiplied by the number of donor bacterial colonies (in CFU per milliliter). The data were determined in three independent experiments, and differences were deemed significant when the *P* value was <0.05 by Student’s *t* test.

### Susceptibility of strains to antibiotics.

The MICs of olaquindox for ST258, ST258/pKpQIL-UK, and ST258/pKpQIL-D2 were determined using the guidelines recommended by the British Society of Antimicrobial Chemotherapy (100). E. coli NCTC 10418 was used as the control strain.

### Biofilm formation.

The impact of plasmid carriage on biofilm formation was assessed in 96-well microtiter trays as previously described (101). The ability of each strain, with and without a plasmid, to form a biofilm was determined in three independent experiments. For Ecl8 strains, temperatures of 37, 30, and 22°C were used; for ST258 strains, temperatures of 37 and 30°C were used. Data were analyzed using Student’s *t* test, and differences represented by *P* values of <0.05 were considered significant. Biofilm formation was also assessed at 22, 30, and 37°C under conditions of constant flow of liquid media for 48 h in glass microfluidic flow cells as previously described (101). The biofilms were observed by phase-contrast microscopy at 6-, 12-, 24-, and 48-h time points at ×40 magnification. The area of coverage by the biofilm in the microfluidic channel was determined using ImageJ (https://imagej.nih.gov/ij/). Student’s *t* test was used to determine whether significant differences (*P* < 0.05) were observed.

### Plasmid persistence.

The proportion of the bacterial population that retained a plasmid was determined over a period of 20 days as previously described (69). Bacteria were subcultured into fresh broth without antibiotics at a dilution of 1 in 100 daily for 20 days. At days 5, 10, 15, and 20, the culture was diluted and plated on agar and incubated overnight at 37°C. Colonies were replica plated on agar supplemented with 0.25 mg/liter doripenem. Plasmid retention was calculated as the percentage of doripenem-resistant colonies in the total number of colonies on the antibiotic-free LB agar. The experiment was repeated on three separate occasions. Whole-genome sequencing was not performed after the 20-day persistence experiments.

### Pairwise competition.

Single colonies of K. pneumoniae Ecl8^Rif^ strains carrying pKpQIL-UK and pKpQIL-D2 *bla*_KPC_::*aph* were inoculated into 10 ml of broth supplemented with 0.25 mg/liter doripenem and were incubated overnight at 37°C at 200 rpm. These were diluted with fresh LB broth to an optical density at 600 nm (OD_600_) of 0.1. Then, equal volumes of the bacterial suspensions were used to inoculate 10 ml of fresh antibiotic-free broth and incubated overnight at 37°C at 200 rpm. Each day for 20 days, the culture was subcultured into 10 ml of fresh broth. To determine the ratio of plasmid-carrying strains within the population, samples were removed at days 5, 10, 15, and 20, diluted, plated on agar, and incubated. The following day, colonies were replica plated as described above onto 2 LB plates, one supplemented with 0.25 mg/liter doripenem and the other with 50 mg/liter kanamycin. The competition experiment was repeated on three separate occasions. The competition index of the bacterial strains was calculated using the following formula (102):

$$\text{Competition Index }=\;\frac{X\;-\;Y}{X\;+\;Y}$$

where *X* represents the average proportion of pKpQIL-D2-carrying K. pneumoniae Ecl8^Rif^ or ST258 in the total bacterial population and *Y* represents the equivalent for the pKpQIL-UK-carrying strain at the end of the experiment. A positive value indicates that a strain carrying the pKpQIL-D2 plasmid had an advantage over the pKpQIL-UK-carrying strain. A negative value indicates that the presence of the pKpQIL-D2 plasmid was disadvantageous.

### G. mellonella infection.

The CFU counts per milliliter for the overnight cultures of the K. pneumoniae ST258 and the pKpQIL-UK and pKpQIL-D2-carrying strains were determined by counting of viable cells. The Galleria mellonella Wax Moth larvae were purchased from Livefood UK Ltd. Larvae of 2 cm in length were placed in groups of 10 into sterile petri dishes. The overnight cultures of the bacterial strains were diluted in sterile phosphate-buffered saline (PBS) to obtain an inoculum of 10^7^ CFU/ml. A total of 10^5^ CFU in 10 µl was injected into each larva at the last right proleg. The larvae were incubated in the dark at 37°C. The percentage of survival of the larvae was recorded every 24 h over a period of 5 days. A larva was considered dead when it was black in color and when no movement was observed when gently agitated. Larvae which were black in color but still moving or which were not moving but had not undergone the color change were not considered dead. The experiment was repeated on two separate occasions. Kaplan-Meier survival curves were plotted using GraphPad Prism, and the log rank (Mantel-Cox) test was used to determine whether significant differences (*P* < 0.05) in survival were observed between the groups of larvae infected with the different strains.

### RNA sequencing.

K. pneumoniae ST258, ST258/pKpQIL-UK, and ST258/pKpQIL-D2 were grown in MOPS minimal medium (Teknova) at 37°C with shaking at 200 rpm. The strains were then subcultured into fresh MOPS minimal medium the next day at a dilution of 1 in 50 and allowed to grow until the OD_600_ reached 0.6. RNA was extracted using a RiboPure RNA purification kit (Ambion) according to the manufacturer’s instructions. The quality of the DNase-treated RNA samples was analyzed using a 2100 Bioanalyzer (Agilent, CA) with an RNA 6000 Nano kit (Agilent). Samples were purified using an RNA Clean and Concentrator-5 kit (Zymo Research). The rRNA was depleted using a Ribo-Zero rRNA removal kit (Gram-negative bacteria) (Epicentre) according to the manufacturer’s instructions. The rRNA-depleted RNA samples were concentrated using an RNA Clean and Concentrator-5 kit and RNase-free water. Then, the samples were analyzed using a 2100 Bioanalyzer (Agilent, CA) with an RNA 6000 Pico kit (Agilent). The library preparation was performed using a TruSeq stranded mRNA sample preparation kit (Illumina). The quality of each library was determined using a model 2200 TapeStation (Agilent) with a D1000 ScreenTape kit (Agilent). The libraries were quantified using a Kapa library quantification kit for next-generation sequencing (KAPA Biosystems). The sequencing was done using a MiSeq sequencer (Illumina) and MiSeq reagent kit v3 (Illumina). The sequencing parameters were set at 200-bp paired-end reads and 400 cycles.

Raw sequences were quality assessed using FASTQC and processed. Alignments were performed using bowtie2 (103). A bed file of the gene loci was generated from GFF annotations, and BEDTools was used to count the tags overlapping the regions of interest (104). Raw tag counts per sample were scale normalized to the count determined for the sample with the lowest number of tags within each data set. Count values were converted to log_2_ values and subjected to quantile normalization within each series for comparisons within each data set. Pairwise comparisons were performed using the normalized tag counts and linear modeling (Bioconductor limma package) (105). A raw cutoff value of 0.05 was used to produce a list of changed genes. To ensure that potentially biologically relevant changes were not overlooked, fold change cutoff determinations were not used. RNA sequencing data were submitted to NCBI GEO. Gene annotation was performed using PROKKA (http://www.vicbioinformatics.com/software.prokka.shtml) (106), and the data were compared with annotations that had been previously published (33).

### Whole-genome sequencing.

After the plasmids were inserted into the strains and were confirmed by PCR, DNA was extracted from strains ST258, ST258/pKpQIL-D2, ST258/pKpQIL-UK, Ecl8, Ecl8/pKpQIL-D2, and Ecl8/pKpQIL-UK using a bacterial genomic DNA isolation kit (Norgen Corp.; catalog no. 17900). WGS was performed by the Beijing Genomics Institute (BGI), using paired-end sequencing on an Illumina HiSeq 4000 platform. *De novo* assemblies of the plasmid-free parent strains were performed using SPAdes with the –careful flag and were annotated using Prokka. Raw sequence data of plasmid-containing strains were aligned to the sequence of the plasmid-free parent using Breseq, with reads mapped against each individual contig of the *de novo* assembly to avoid issues associated with contig breaks. In order to look for SNPs and indels between the plasmid DNA sequences of pKpQIL-D2 (KY798506), ST258/pKpQIL-D2, and Ecl8/pKpQIL-D2 and of pKpQIL-UK (KY798507), ST258/pKpQIL-UK, and Ecl8/pKpQIL-UK, the snippy program was used (https://github.com/tseemann/snippy).

### Accession number(s).

WGS data have been deposited in the European Nucleotide Archive (ENA) project under accession number PRJEB23315.
