# Supplementary material for: Clinically Relevant Plasmid-Host Interactions Indicate that Transcriptional and Not Genomic Modifications Ameliorate Fitness Costs of Klebsiella pneumoniae Carbapenemase-Carrying Plasmids
Source: mBio. 2018 Apr 24;9(2):e02303-17. doi: 10.1128/mBio.02303-17 (PMC5915730; doi:10.1128/mBio.02303-17)
Supplement: FIG S1 [file mbo002183847sf1.pdf]

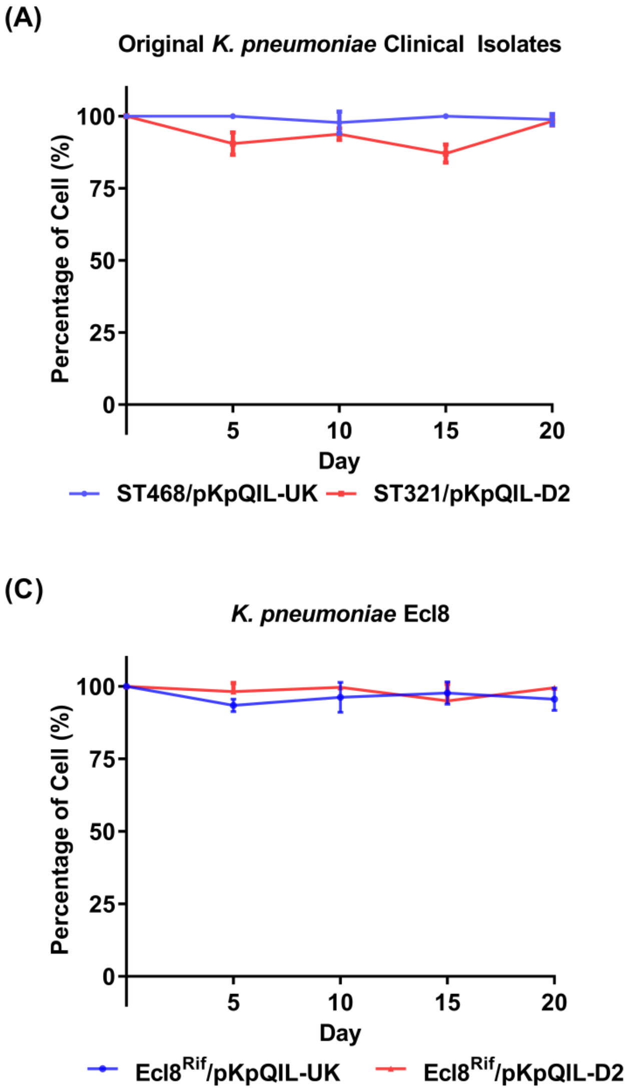

Figure S1. The percentage of pKpQIL-UK vs -D2 carrying cells over a period of 20 days without antibiotic selection. The persistence of the plasmids pKpQIL-UK (blue) and pKpQIL-D2 (red) in *K. pneumoniae* (A) ST468/pKpQIL-UK, ST321/pKpQIL-D2, (B) ST258, and (C) Ecl8 all in LB broth without antibiotic selection. The percentages of cells which retain the plasmids were recorded as mean ± standard deviation of three independent experiments.
