# Supplementary material for: Clinically Relevant Plasmid-Host Interactions Indicate that Transcriptional and Not Genomic Modifications Ameliorate Fitness Costs of Klebsiella pneumoniae Carbapenemase-Carrying Plasmids
Source: mBio. 2018 Apr 24;9(2):e02303-17. doi: 10.1128/mBio.02303-17 (PMC5915730; doi:10.1128/mBio.02303-17)
Supplement: FIG S2 [file mbo002183847sf2.pdf]

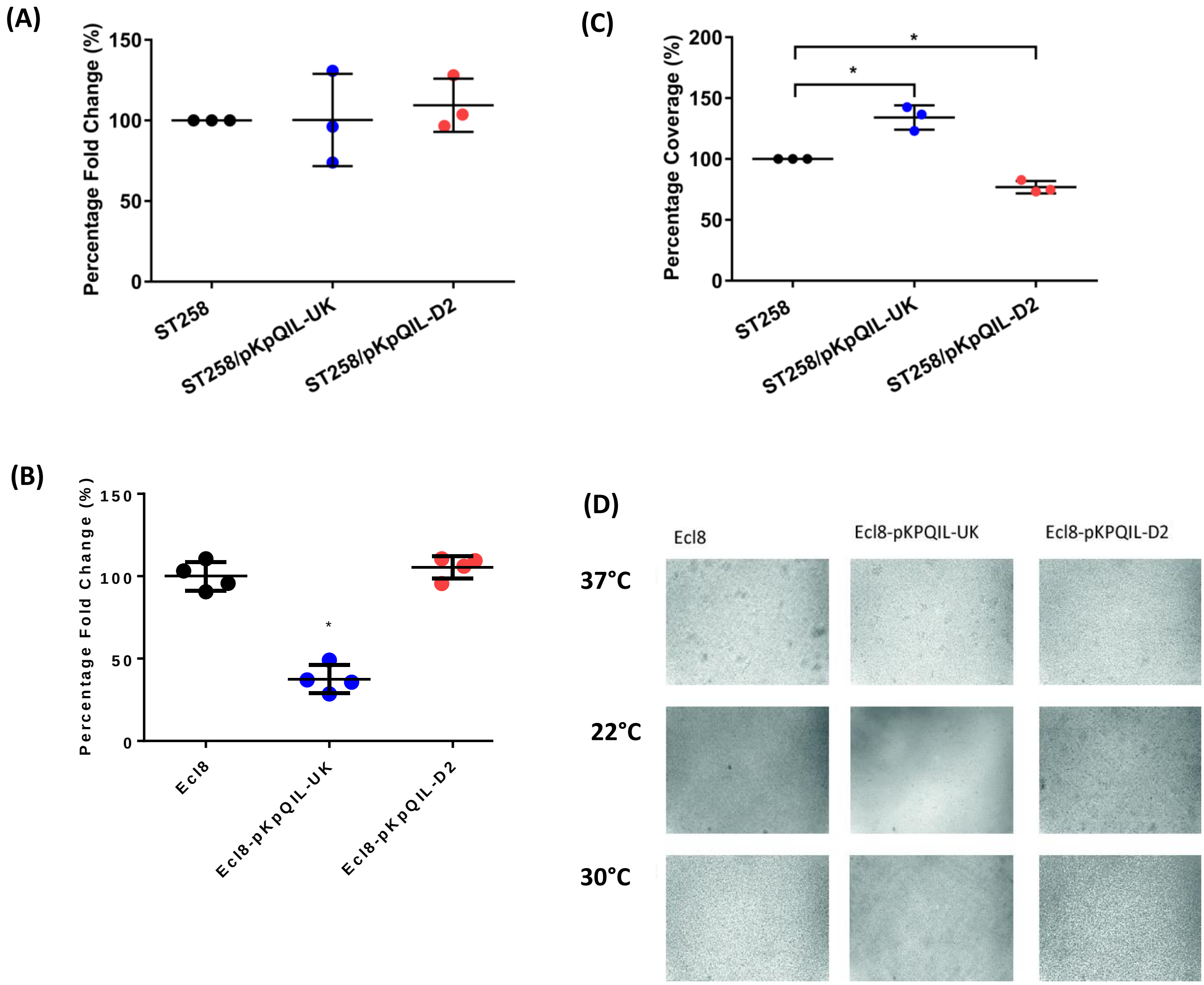

Figure S2. Biofilm formation (a) on plastic for ST258 at 30°C and (b) on plastic for Ecl8 at 22°C. The impact of the plasmids pKpQIL-UK (blue) and pKpQIL-D2 (red) on its hosts' ability to form biofilm was investigated using the microtiter tray and crystal violet dye method. Percentage fold change was recorded as mean ± standard deviation of a minimum of three independent experiments. (c) Percentage of coverage by biofilm in microfluidic channel after 48 hours under constant flow of LB broth by the pKpQIL-UK (blue) and pKpQIL-D2 (red) carrying *K. pneumoniae* ST258 on the surface of the microfluidic channel was estimated using ImageJ software. The percentage coverage relative to the parental strain was recorded as mean ± standard deviation of three independent experiments. (d) Biofilm growth of Ecl8, Ecl8/pKpQIL-UK, and Ecl8/pKpQIL-D2 in a fluidic channel under constant flow at 37, 30, and 22°C. Representative images of three independent experiments. Student's *t*-test was used to analyse significant changes which are shown with asterisk (*) $p<0.05$.
