# Supplementary material for: Clinically Relevant Plasmid-Host Interactions Indicate that Transcriptional and Not Genomic Modifications Ameliorate Fitness Costs of Klebsiella pneumoniae Carbapenemase-Carrying Plasmids
Source: mBio. 2018 Apr 24;9(2):e02303-17. doi: 10.1128/mBio.02303-17 (PMC5915730; doi:10.1128/mBio.02303-17)
Supplement: FIG S3 [file mbo002183847sf3.pdf]

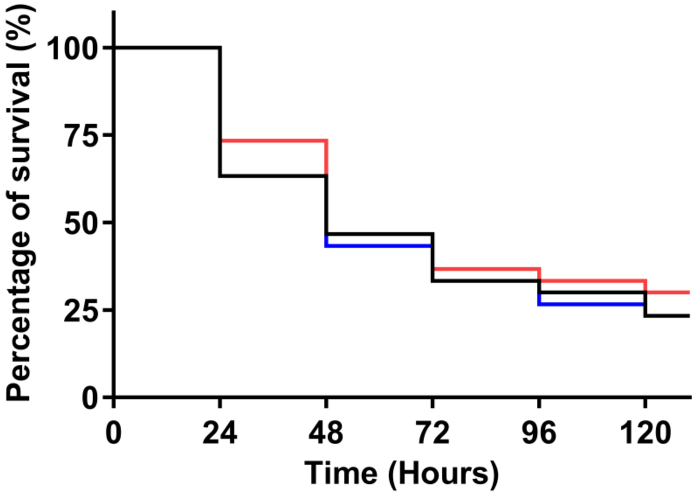

Figure S3. Virulence assay of *Galleria mellonella* infected by *K. pneumoniae* ST258 carrying either pKpQIL-UK (blue) or pKpQIL-D2 (red). Percentage of *G. mellonella* that survived after infection was recorded as mean ± standard deviation of three independent experiments at 24-hour intervals for five days. Each experiment consisted of a group of 10 *Galleria* larvae. Log-rank (Mantel-Cox) test was used to analyse the differences in survival of the two populations infected with plasmid-carrying *K. pneumoniae* ST258 compared with the wild-type host.
